# Supplementary material for: Towards Quantitative and Standardized Serological and Neutralization Assays for COVID-19
Source: Int J Mol Sci. 2021 Mar 8;22(5):2723. doi: 10.3390/ijms22052723 (PMC7962843; doi:10.3390/ijms22052723)
Supplement: Supplementary file 1 [file ijms-22-02723-s001.pdf]

Supplemental Information

Figure S1. Each viral antigen listed in the table was coupled to a microbead population with a distinct address, e.g., R26 for SARS-CoV-2 RBD that displayed a different fluorescence intensity in the fluorescence channel, R712-APC-700 of the CytoFLEX cytometer (right plot). Because the serology assays and assays assessing cross reactivity were performed separately in different sample wells, assay performance was not affected by the overlap of the intensity addresses of R51 (spike) and R56 (SARS).

MagPlex-C beads	R712-APC-700
R26 (SARS-CoV-2 RBD, blue)	✓
R47 (MERS, orange)	✓
R51 (SARS-CoV-2 Spike, green)	✓
R56 (SARS, dark green)	✓
R62 (OC43, light purple)	✓
R77 (HKU1, purple)	✓

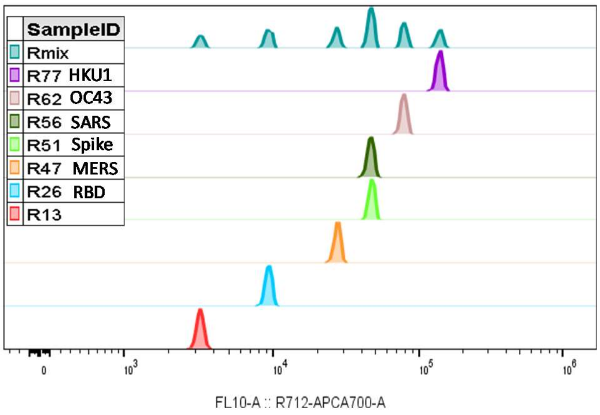

Figure S2. A plot of sample dilution vs. reduction % is shown for a positive convalescent patient sample as an example. The NT_{50} (grey dot) was determined by modeling the does-response curve shown as red line using Eq. 4 provided in the figure.

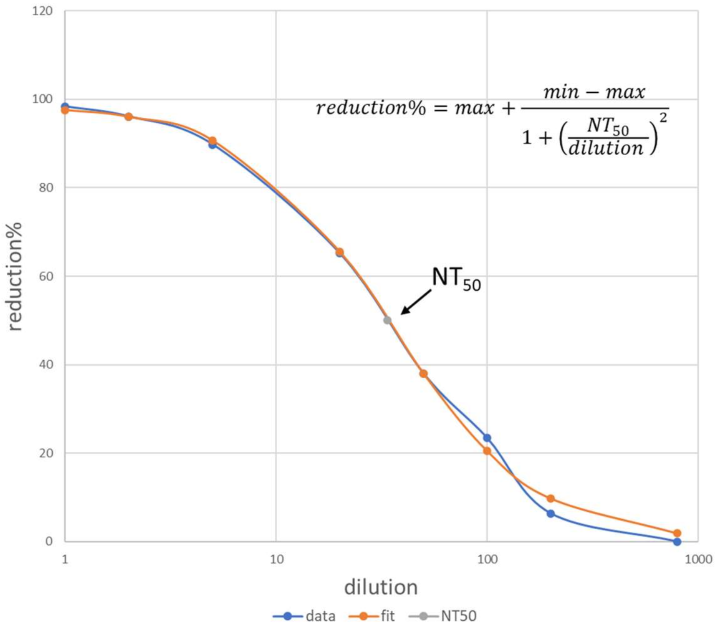

Supplemental coronaviruses spike protein sequences: Comparison of the full length spike protein sequence for R619-X16: SARS-CoV2 S-2P-3C-His8-Strep2x2 (SARS-COV2), R619-X17: SARS-CoV S-2P-3C-His8-Strep2 (SARS-CoV), R619-X18: MERS-CoV S-2P-3C-His8-Strep2x2 (MERS-CoV), R619-X19: OC43 S-2P-3C-His8-Strep2x2 (OC43), and R619-X20: HKU1 S-2P-3C-His8-Strep2 (HKU1). The S1 section is in blue text, the RBD section green text and the S2 section purple text. The highlights indicate the homology compared with SARS-Cov2. Blue indicates all five sequences have the same the amino acid, pink indicates four of the five sequences, green for three and yellow for two sequences the same. Abbreviations: 3C, rhinovirus 3C protease cleavage site; Strep2x2, dual Strep2 epitope tag; T7, bacteriophage T7 fibritin trimerization domain; SBP, streptavidin binding peptide. The sequence alignment was carried out using Clustal Omega program (https://www.ebi.ac.uk/Tools/msa/clustalo/).

```
                              S1
SARS-CoV2         ------------CVNLT---------TRTQL---PPAYTNSFTRGVYYPDKVFRSSVLHS 36
SARS-CoV          -------SDLDRCTTFD---------DVQAP---NYTQHTSSMRGVYYPDEIFRSDTLYL 41
MERS-CoV          YVDVGPDSVKSACIEVDIQQT---FFDKTWP---R-PIDVSKADGIIYPQGRTYSNITIT 53
OC43              --VIG----DLKCPL-DSRTGSLNNIDTGPPSISTATVDVTNGLGTYYVLDRVYLNTTLF 53
HKU1              --VIG----DFNCTN-----SFINDYNKTIPRISEDVVDVSLGLGTYYVLNRVYLNTTLL 49

SARS-CoV2         TQDLFLPFFSNVT---WFHAIHVSGTNGTKR----FDNP-VLPFNDGVYFAST------- 81
SARS-CoV          TQDLFLPFYSNVT---GFHTIN-------HT----FGNP-VIPFKDGIYFAAT------- 79
MERS-CoV          YQGLF-PYQGDHGDMYVYSAGHATGTTPQKLFVANYSQD-VKQFANGFVVRIGAAANSTG 111
OC43              LNGYYPTSGSTYRNMA------LKGTDKLS--TLWFKPPFLSDFINGIFAKVKNTKVFKD 105
HKU1              FTGYFPKSGANFRDLA------LKGSIYLS--TLWYKPPFLSDFNNGIFSKVKNTKLYVN 101

SARS-CoV2         ------E----KSNIIRGWIFGTTLDSKTQ---------SLLIVNN-------A-----T 110
SARS-CoV          ------E----KSNVVRGWVFGSTMNNKSQ---------SVIIINN-------S-----T 108
MERS-CoV          TVIISPSTSATIRKIYPAFMLGSSVGNFSDGKMGRFFNHTLVLLPD-------G-----C 159
OC43              GV---------MYSEFPAITIGSTFVNTSY---------SVVVQPRTINSTQDGVNKLQG 147
HKU1              NT---------LYSEFSTIVIGSVFVNTSY---------TIVVQPH------------NG 131

SARS-CoV2         NVVIKVCEFQFCNDPFLGVYYHKN---------------------NKSWMESEFRVYSSA 149
SARS-CoV          NVVIRACNFELCDNPFFAVSKPMG---------------------T----QTHTMIFDNA 143
MERS-CoV          GTLLRAF--YCILEPRSGNHCPAGNSYTSFATYHTPATDCSDGNYNRNASLNSFKEYFNL 217
OC43              LLEVSVCQYNMCEYPHTICHPKLGNHF------------------KELWHMDTGV-VSCL 188
HKU1              ILEITACQYTMCEYPHTVCKSK-GSIR------------------NESWHIDSSE-PLCL 171

SARS-CoV2         NNCTFEYVSQPFLMDLEGKQGNFKNLREFVFKNIDGYFKIYSKHTPINLVRDLPQGFSAL 209
SARS-CoV          FNCTFEYISDAFSLDVSEKSGNFKHLREFVFKNKDGFLYVYKGYQPIDVVRDLPSGFNTL 203
MERS-CoV          RNCTFMYTYNITEDEILEWFGITQTAQG-VHLFSSRYVDLYGGN---------------M 261
OC43              YKRNFTYDVNATYL-------------YFHFYQEGGTFYAYFTDT--GVV---------T 224
HKU1              FKKNFTYNVSADWL-------------YFHFYQERGVFYAYYADV--GMP---------T 207

SARS-CoV2         EPLVDLPIGINITRFQTLLALHRSYLTPGDSSSGWTAGAAAYYVGYLQPRTFLLKYNENG 269
SARS-CoV          KPIFKLPLGINITNFRAILTAFS------PAQDIWGTSAAAYFVGYLKPTTFMLKYDENG 257
MERS-CoV          FQFATLPVYDTIKYYSIIPHSIR---SIQSDRKAW----AAFYVYKLQPLTFLLDFSVDG 314
OC43              KFLFNVYLGMALSHYYVMPLTCI---S--R---RDIGFTLEYWVTPLTSRQYLLAFNQDG 276
HKU1              TFLFSLYLGTILSHYYVMPLTCN---AISS---NTDNETLEYWVTPLSRRQYLLNFDEHG 261

                                                        RBD
```

```
SARS-CoV2   TITDAVDCALDPLSETKCTLKSFTVEKGIYQTSNFR VQPTESIVRFPNITNLCPFGEVFN 329
SARS-CoV    TITDAVDCSQNPLAELKCSVKSFEIDKGIYQTSNFR VVPSGDVVRFPNITNLCPFGEVFN 317
MERS-CoV    YIRRAIDCGFNDLSQLHCSYESFDVESGVYSVSSFE AKPSGSVVEQAE-GVECDFSPLLS 373
OC43        IIFNAVDCMSDFMSEIKCKTQSIAPPTGVYELNGYT VQPIADVYRRKPDLPNCNIEAWLN 336
HKU1        VITNAVDCSSSFLSEIQCKTQSFAPNTGVYDLSGFT VKPVATVYRRIPNLPDCDIDNWLN 321

SARS-CoV2   ATRFASVYAWNRKRISNCVADYSVLYNSASFSTFKCYGVSPTKLNDLCFTNVYADSFVIR 389
SARS-CoV    ATKFPSVYAWERKKISNCVADYSVLYNSTFFSTFKCYGVSATKLNDLCFSNVYADSFVVK 377
MERS-CoV    G-TPPQVYNFKRLVFTNCNYNLTKLLSLFSVNDFTCSQISPAAIASNCYSSLILDYFSYP 432
OC43        DKSVPSPLNWERKTFSNCNFNMSSLMSFIQADSFTCNNIDAAKIYGMCFSSITIDKFAIP 396
HKU1        NVSVPSPLNWERRIFSNCNFNLSTLLRLVHVDSFSCNNLDKSKIFGSCFNSITVDKFAIP 381

SARS-CoV2   GDEVRQIAPGQTGKIADYNYKLPDDFTGCVIAWNSNNLDSKVGGNYNYLYRLFRKSNL-- 447
SARS-CoV    GDDVRQIAPGQTGVIADYNYKLPDDFMGCVLAWNTRNIDATSTGNYNYKYRYLRHGKL-- 435
MERS-CoV    LSMKSDLSVSSAGPISQFNYKQSFSNPTCLILATVPHNLTTITKPLKYSYINKCSRFL-- 490
OC43        NGRKVDLQLGNLGYLQSFNYRIDTTATSCQLYYNLPAANVSVSR-FNPSTWNKRFGFIEN 455
HKU1        NRRRDDLQLGSSGFLQSSNYKIDISSSSCQLYYSLPLVNVTINN-FNPSSWNRRYGFGSF 440

SARS-CoV2   ---KPFER--------DISTEIYQ--AGSTPCNGV-----------------EG-----F 472
SARS-CoV    ---RPFER--------DISNVPFS--PDGKPCTP------------------PA-----L 459
MERS-CoV    ---SDDRT--------EVPQLVNA--NQYSPCVSI-----------------VP-----S 515
OC43        SVFKPQPAGVLTNHDVVYAQHCFKAPKNFCPCKLNSSLCVGSGPGKNNGIGTCPAGTNYL 515
HKU1        N---------LSSYDVVYSDHCFSVNSDFCPCADPSVVN--SCAKSKPPSAICPAGTKYR 489

SARS-CoV2   NCYF-----------------PLQSYGFQ-----------------------------PT 486
SARS-CoV    NCYW-----------------PLNDYGFY-----------------------------TT 473
MERS-CoV    T-VWEDGDYYRK------QLSPLEGGGWL-----------------------------VA 539
OC43        TC------------HNLCNPDPITFTGPYKCPQTKSLVGIGEHCSGLAVKSDYCGG---- 559
HKU1        HCDLDTTLYVKNWCRCSCLPDPISTYSPNTCPQKKVVVGIGEHCPGLGINEEKCGTQLNH 549

SARS-CoV2   NGVGYQPYRVVVLSFEL------------L----HAPATVCGP-----KKSTNLVKNKCV 525
SARS-CoV    TGIGYQPYRVVVLSFEL------------L----NAPATVCGP-----KLSTDLIKNQCV 512
MERS-CoV    SGSTVAMTEQLQMGFGI------------TVQYGTDTNSVCPKLEFANDTKIASQLGNCV 587
OC43        NPCTCQPQAFLGWSADSCLQGDKCNIFANLILHDVNSGLTCSTD--LQKANTDIKLGVCV 617
HKU1        SSCFCSPDAFLGWSFDSCISNNRCNIFSNFIFNGINSGTTCSND--LLYSNTEISTGVCV 607

           End of RBD
SARS-CoV2   NF NFNGLTGTGVLTESNKKF-LPFQQFGRDIADTT-DAVRDPQTLEILDITPCSFGGVSV 583
SARS-CoV    NF NFNGLTGTGVLTPSSKRF-QPFQQFGRDVSDFT-DSVRDPKTSEILDISPCAFGGVSV 570
MERS-CoV    EY SLYGVSGRGVFQNCTAVG-VRQQRFVYDAYQNLVGYYSD--DGNYYCLRACVSVPVSV 644
OC43        NY DLYGISGQGIFVEVNATYYNSWQNLLYDSNGNLY-GFRDYITNRTFMIRSCYSGRVSA 676
HKU1        NY DLYGITGQGIFKEVSAAYYNNWQNLLYDSNGNII-GFKDFLTNKTYTILPCYSGRVSA 666

SARS-CoV2   ITPGTNTSNQVAVLYQDVNCTEVPVAIHADQLT--PTWRVYSTGSNVFQTRAGCLIGAEH 641
SARS-CoV    ITPGTNASSEVAVLYQDVNCTDVSTAIHADQLT--PAWRIYSTGNNVFQTQAGCLIGAEH 628
MERS-CoV    IYD--KETKTHATLFGSVACEHISSTMSQYSRSTRSMLKRRDSTYGPLQTPVGCVLGLVN 702
OC43        AFH--ANSSEPALLFRNIKCNYVFNNSLIRQLQ----------PINYFDSYLGCVVNAYN 724
HKU1        AFY--QNSSSPALLYRNLKCSYVLNNIS--FIS----------QPFYFDSYLGCVLNAVN 712

                                                 beginning of S2
SARS-CoV2   VN--NSYECDIPIGAGICASYQTQTNSP-GSAS SVAS---QS-IIAYTMSLGA-ENSVAY 693
SARS-CoV    VD--TSYECDIPIGAGICASYHTVSLL-----R STSQ---KS-IVAYTMSLGA-DSSIAY 676
MERS-CoV    SS-LFVEDCKLPLGQSLCALPDTPSTLTPASVG SVPGEMRLA-SIAFNHPIQV--DQLNS 758
OC43        STAISVQTCDLTVGSGYCVDYSKNR--RSRRAI TTGY--RFTNFEPFTVNSVNDSLEPVG 780
HKU1        LTSYSVSSCDLRMGSGFCIDYALPSSGGSGSGI SSPY--RFVTFEPFNVSFVNDSVETVG 770

SARS-CoV2   SNNSIAIPTNFTISVTTEILPVSMTKTSVDCTMYICGDSTECSNLLLQYGSFCTQLNRAL 753
SARS-CoV    SNNTIAIPTNFSISITTEVMPVSMAKTSVDCNMYICGDSTECANLLLQYGSFCTQLNRAL 736
MERS-CoV    SYFKLSIPTNFSFGVTQEYIQTTIQKVTVDCKQYVCNGFQKCEQLLREYGQFCSKINQAL 818
OC43        GLYEIQIPSEFTIGNMEEFIQTSSPKVTIDCAAFVCGDYAACKSQLVEYGSFCDNINAIL 840
HKU1        GLFEIQIPTNFTIAGHEEFIQTSSPKVTIDCSAFVCSNYAACHDLLSEYGTFCDNINSIL 830
```

```
SARS-CoV2     TGIAVEQDKNTQEVFAQVKQIYKTPP-----IKDFGGF-NFSQILP-DP---SKPSKRSF  803
SARS-CoV      SGIAAEQDRNTREVFAQVKQMYKTPT-----LKYFGGF-NFSQILP-DP---LKPTKRSF  786
MERS-CoV      HGANLRQDDSVRNLFASVKSSQSSPI-----IPGFGGDFNLTLLEP-VSISTGSRSARSA  872
OC43          TEVNELLDTTQLQVANSLMNGVTLSTKLKDGVNFNVDDINFSSVLGCLGSECSKASSRSA  900
HKU1          NEVNDLLDITQLQVANALMQGVTLSSNLNTNLHSDVDNIDFKSLLGCLGSQCGS-SSRSL  889

SARS-CoV2     IEDLLFNKVTLADAGFIKQYGDCL--GDIAARDLICAQKFNGLTVLPPLLTDEMIAQYTS  861
SARS-CoV      IEDLLFNKVTLADAGFMKQYGECL--GDINARDLICAQKFNGLTVLPPLLTDDMIAAYTA  844
MERS-CoV      IEDLLFDKVTIADPGYMQGYDDCMQQGPASARDLICAQYVAGYKVLPPLMDVNMEAAYTS  932
OC43          IEDLLFDKVKLSDVGFVAAYNNCT--GGAEIRDLICVQSYKGIKVLPPLLSENQISGYTL  958
HKU1          LEDLLFNKVKLSDVGFVEAYNNCT--GGSEIRDLLCVQSFNGIKVLPPILSETQISGYTT  947

SARS-CoV2     ALLAGTITSGWTFGAGAALQIPFAMQMAYRFNGIGVTQNVLYENQKLIANQFNSAIGKIQ  921
SARS-CoV      ALVSGTATAGWTFGAGAALQIPFAMQMAYRFNGIGVTQNVLYENQKQIANQFNKAISQIQ  904
MERS-CoV      SLLGSIAGVGWTAGLSSFAAIPFAQSIFYRLNGVGITQQVLSENQKLIANKFNQALGAMQ  992
OC43          AATSASLFPPWTAA----AGVPFYLNVQYRINGLGVTMDVLSQNQKLIANAFNNALDAIQ  1014
HKU1          AATVAAMFPPWSAA----AGVPFSLNVQYRINGLGVTMDVLNKNQKLIANAFNKALLSIQ  1003

SARS-CoV2     DSLSSTASALGKLQDVVNQNAQALNTLVKQLSSNFGAISSVLNDILSRLDPPEAEVQIDR  981
SARS-CoV      ESLTTTSTALGKLQDVVNQNAQALNTLVKQLSSNFGAISSVLNDILSRLDPPEAEVQIDR  964
MERS-CoV      TGFTTTNEAFHKVQDAVNNNAQALSKLASELSNTFGAISASIGDIIQRLDPPEQDAQIDR  1052
OC43          EGFDATNSALVKIQAVVNANAEALNNLLQQLSNRFGAISSSLQEILSRLDPPEAEAQIDR  1074
HKU1          NGFTATNSALAKIQSVVNANAQALNSLLQQLFNKFGAISSSLQEILSRLDPPEAQVQIDR  1063


SARS-CoV2     LITGRLQSLQTYVTQQLIRAAEIRASANLAATKMSECVLGQSKRVDFCGKGYHLMSFPQS  1041
SARS-CoV      LITGRLQSLQTYVTQQLIRAAEIRASANLAATKMSECVLGQSKRVDFCGKGYHLMSFPQA  1024
MERS-CoV      LINGRLTTLNAFVAQQLVRSESAALSAQLAKDKVNECVKAQSKRSGFCGQGTHIVSFVVN  1112
OC43          LINGRLTALNAYVSQQLSDSTLVKFSAAQAMEKVNECVKSQSSRINFCGNGNHIISLVQN  1134
HKU1          LINGRLTALNAYVSQQLSDITLIKAGASRAIEKVNECVKSQSPRINFCGNGNHILSLVQN  1123

SARS-CoV2     APHGVVFLHVTYVPAQEKNFTTAPAICHDGKA---HFPREGVFVSN-----GTHWFVTQR  1093
SARS-CoV      APHGVVFLHVTYVPSQERNFTTAPAICHEGKA---YFPREGVFVFN-----GTSWFITQR  1076
MERS-CoV      APNGLYFMHVGYYPSNHIEVVSAYGLCDAANPTNCIAPVNGYFIKTNNTRIVDEWSYTGS  1172
OC43          APYGLYFIHFSYVPTKYVTAKVSPGLCIAGDR--GIAPKSGYFVNV-----NNTWMYTGS  1187
HKU1          APYGLLFIHFSYKPTSFKTVLVSPGLCLSGDR--GIAPKQGYFIKQ-----NDSWMFTGS  1176

SARS-CoV2     NFYEPQIITTDNTFVSGNCDVVIGIVNNTVYDPLQ--PELDSFKEELDKYFKNHTSPDVD  1151
SARS-CoV      NFFSPQIITTDNTFVSGNCDVVIGIINNTVYDPLQ--PELDSFKEELDKYFKNHTSPDVD  1134
MERS-CoV      SFYAPEPITSLNTKYVAPQVTYQN-ISTNLPPPLLGNSTGIDFQDELDEFFKNVSTSIPN  1231
OC43          GYYYPEPITENNVVVMSTCAVNYTKAPYVML--NTSTPNLPDFREELDQWFKNQTSVAPD  1245
HKU1          SYYYPEPISDKNVVFMNSCSVNFTKAPFIYL--NNSIPNLSDFEAELSLWFKNHTSIAPN  1234

SARS-CoV2     LGDISGINASVVNIQKEIDRLNEVAKNLNESLIDLQELGKYEQGSGYIPEAPRDGQAYVR  1211
SARS-CoV      LGDISGINASVVNIQKEIDRLNEVAKNLNESLIDLQELGKYEQGSGYIPEAPRDGQAYVR  1194
MERS-CoV      FGSLTQINTTLLDLTYEMLSLQQVVKALNESYIDLKELGNYTYGSGYIPEAPRDGQAYVR  1291
OC43          LSLD-YINVTFLDLQVEMNRLQEAIKVLN--------------GSGYIPEAPRDGQAYVR  1290
HKU1          LTFNSHINATFLDLYYEMNVIQESIKSLN--------------GSGYIPEAPRDGQAYVR  1280

SARS-CoV2     KDGEWVLLSTFLGRSLEVLFQGPGHHHHHHHHSAWSHPQFEKGGGSGGGGSGGSAWSHPQ  1271
SARS-CoV      KDGEWVLLSTFLGRSLEVLFQGPGHHHHHHHHSAWSHPQFEK------------------  1236
MERS-CoV      KDGEWVLLSTFLGRSLEVLFQGPGHHHHHHHHSAWSHPQFEKGGGSGGGGSGGSAWSHPQ  1351
OC43          KDGEWVLLSTFLGRSLEVLFQGPGHHHHHHHHSAWSHPQFEKGGGSGGGGSGGSAWSHPQ  1350
HKU1          KDGEWVLLSTFLGRSLEVLFQGPGHHHHHHHHSAWSHPQFEK------------------  1322

SARS-CoV2     FEK        1274
SARS-CoV      ---        1236
MERS-CoV      FEK        1354
OC43          FEK        1353
HKU1          ---        1322
```
